# Efficacy and Safety of Laser Therapy for the Treatment of Genitourinary Syndrome of Menopause: A Protocol for Systematic Review and Meta-Analysis of Clinical Trials

**DOI:** 10.3389/frph.2021.772690

**Published:** 2021-10-26

**Authors:** Lisieux de Lourdes Martins Nóbrega Pessoa, Ayane Cristine Alves Sarmento, Kleyton Santos Medeiros, Ana Paula Ferreira Costa, Ana Katherine Gonçalves, Ricardo Ney Cobucci

**Affiliations:** ^1^Graduate Program in Sciences Applied to Women's Health, Maternity School Januário Cicco (MEJC), Federal University of Rio Grande do Norte (UFRN), Natal, Brazil; ^2^Postgraduate Program in Health Sciences, Federal University of Rio Grande do Norte (UFRN), Natal, Brazil; ^3^Department of Obstetrics and Gynaecology, Federal University of Rio Grande do Norte (UFRN), Natal, Brazil; ^4^Graduate Program of Biotechnology, Potiguar University (UnP), Natal, Brazil

**Keywords:** post-menopausal women, laser therapy, atrophy, urinary incontinence, systematic review

## Abstract

Laser therapy has been proposed to improve the symptoms of genitourinary syndrome of menopause (GSM), especially in women who do not accept hormonal therapy or are at a high risk of complications if they undergo hormonal therapy. However, studies evaluating the effectiveness and safety of laser treatment for GSM have shown controversial results. Thus, we aimed to determine the efficacy and safety of laser therapy in post-menopausal women with GSM. We have developed a protocol according to the Preferred Reporting Items for Systematic Review and Meta-analysis Protocol using the population, intervention, comparison, outcome, and study design (PICOS) framework for post-menopausal women who have received no treatment, laser therapy, placebo, or vaginal estrogen for GSM. As per our protocol, randomized controlled trials and quasi-randomized trials, regardless of language of publication, will be searched in PubMed, Embase, Scopus, Web of Science, Cochrane Central Register of Controlled Trials, Cumulative Index to Nursing and Allied Health Literature, and clinicaltrials.gov. Gray literature will be searched in Open Gray and Google Scholar. The reference lists will be scanned for additional trials, and the authors will be contacted if necessary. Outcome data reported in a trial registry, even when no published results were available, will be analyzed. The search will be performed using key terms, such as “post-menopausal women,” “menopausal genitourinary syndrome,” “vulvovaginal atrophy,” and “laser therapy.” Two review authors will independently screen the titles and abstracts, while three others will independently evaluate the full text of each study to determine its eligibility for this systematic review (SR). Any disagreement will be resolved through discussion and consensus. Data extraction will be performed independently using a standardized data collection form. Clinical outcomes, including vaginal atrophy, vaginal pH, dryness, dyspareunia, itching, burning, dysuria, urinary frequency, urinary urgency, and urinary incontinence, will be systematically evaluated. We will not perform a separate search for adverse effects; instead, we will consider the adverse effects described in the included studies. Furthermore, we will summarize the effects of dichotomous outcomes as risk ratios with 95% confidence intervals. On the other hand, continuous outcomes will be summarized by expressing treatment effects as a mean difference with standard deviation or as a standardized mean difference when different scales were used to measure the same outcome. We will use the Cochrane Risk of Bias 2 tool for bias assessment and the Grading of Recommendations Assessment, Development and Evaluation approach to rate the overall certainty of evidence. Review Manager 5.3.5 will be used for quantitative data synthesis, subgroup analysis, sensitivity analysis, meta-regression, and risk of bias assessment. The SR findings will provide highly relevant evidence through the synthesis of well-designed and robust clinical trials on the effectiveness and safety of laser therapy in GSM. The Prospective Register of Systematic Reviews (PROSPERO) registration number (2021) of the SR is CRD42021253605.

## Introduction

The genitourinary syndrome of menopause (GSM) affects about 50% of women who undergo menopause. It occurs due to hypoestrogenism and affects the vulvar, vaginal, and urological tissues (1). Vulvovaginal symptoms include vaginal pain, dyspareunia, dryness, itching, and tissue friability, while urological symptoms include urinary frequency, urgency, incontinence, hematuria, and recurrent urinary tract infections (2). The recommended treatment for GSM includes non-hormonal therapies, such as vaginal lubricants and moisturizers, as well as hormonal therapies, such as estrogen-based medications (3). Currently, ospemifene, a non-estrogen selective estrogen receptor modulator, is the only approved, effective, and safe non-hormonal treatment option for GSM. However, it has been associated with estrogenic effects on endometrial tissue as well as systemic hot flush symptoms (4).

Laser therapy was introduced as a non-hormonal option for the treatment of GSM. This therapy works by stimulating the body's mechanism to repair, grow, and heal tissue, thereby facilitating tissue regeneration. The two types of lasers most thoroughly evaluated for their use in GSM treatment are the microablative fractional CO_2_ laser and the Erbium: YAG (Er: YAG) laser (5). GSM treatment with a CO_2_ laser or an Er:YAG laser usually consists of three procedures spaced 4–6 weeks apart (6). The CO_2_ laser uses a gaseous medium at a wavelength of 10,600 nm, which results in various depths of penetration and ablation when absorbed by tissue water (7). On the other hand, the Er: YAG laser uses a solid medium with a wavelength of 2,940 nm, which allows for more focused ablation and deeper thermal secondary effects due to its approximation of the peak of water absorption (8).

The last systematic review (SR) to evaluate laser therapy in GSM treatment concluded that laser therapy for post-menopausal women with GSM appears promising as it can reduce symptom severity, improve quality of life, and restore the vaginal mucosa to its pre-menopausal state. However, the authors claim that the quality of the body of evidence is “low” or “very low.” Thus, evidence-based modifications of the current clinical practice cannot be suggested (2). Recently, another meta-analysis evaluated the effects of laser therapy in women with breast cancer and GSM. The study concluded that large-scale, prospective, randomized controlled trials were necessary to fully explore the benefits of vaginal laser therapy for vaginal atrophy treatment, such as reducing symptom burden and improving the quality of life of post-menopausal women, particularly after breast cancer treatment (1).

Therefore, new studies are needed to assess the effectiveness and safety of lasers in GSM treatment. This protocol proposes a SR with meta-analysis of randomized clinical trials that assess new high-quality evidence supporting laser therapy as a therapeutic option in menopausal women with GSM.

## Materials and Methods

The SR protocol in this study will follow the recommendations of the Preferred Reporting Items for Systematic Review and Meta-analysis Protocol (PRISMA-P) (9). The protocol was registered in the International Prospective Register of Systematic Reviews (PROSPERO) with the registration number CRD42021253605.

### Review Question

The following review question was established: “Is laser therapy an effective and safe option for treating GSM?” The question was formulated based on the PICOS framework, which considers P, population; I, intervention; C, comparison; O, outcome; and S, study design. The elements of the PICOS framework that will be considered for the SR are the following.

- Population/participants: Women with GSM
- Intervention: Laser therapy
- Comparison: No treatment, placebo, vaginal estrogen
- Outcome: vaginal pH, vaginal atrophy, dryness, dyspareunia, itching, burning, dysuria, urinary frequency, urinary urgency, urinary incontinence, urinary tract infections, adverse events, and drop-outs due to adverse events
- Study design: Randomized clinical trials or quasi-randomized clinical trials.

### Eligibility Criteria

Randomized controlled trials and quasi-randomized trials comprising women diagnosed with GSM who were not treated or were undergoing treatment with laser therapy (microablative fractional CO_2_, Er: YAG), placebo, or vaginal estrogen will be included in this study. There will be no restrictions on the search for languages and the publication period.

### Exclusion Criteria

Case reports, observational studies, reviews, letters, preprints, and editorials will be excluded.

### Outcome Measures

Vaginal atrophy will be considered as the primary outcome. It will be evaluated using the vaginal health score index questionnaire (10) that consists of five measures: elasticity, fluid volume, pH, epithelial integrity, and moisture.

#### Secondary Outcomes

The following will be considered and evaluated as secondary outcomes.

- Urinary incontinence will be assessed using micturition diaries, the Urinary Distress Inventory-6 (UDI-6) (11), and the International Consultation on Incontinence Questionnaire-Urinary Incontinence Short Form (ICIQ-UI SF) (12).
- Dyspareunia and dryness will be assessed using the visual analog scale (VAS) 0-10, VAS 0-5, and VAS 0-3 (13).
- Itching, burning, and dysuria will be assessed using the VAS 0-10 (13).
- Frequency and urinary urgency will be assessed using different methodologies, such as micturition diaries, the Overactive Bladder Questionnaire Short Form (OAB-Q SF) (14), the ICIQ- Female Lower Urinary Tract Symptoms (ICIQ-FLUTS) (15), and the UDI-6 (11).
- Urinary tract infections will be assessed using urine culture.
- Adverse events and drop-outs due to adverse events will also be assessed as secondary outcomes.

### Search Strategy

The review authors will search the following databases without language restrictions: PubMed, Embase, Scopus, Web of Science, Cochrane Central Register of Controlled Trials, Cumulative Index to Nursing and Allied Health Literature, and clinical trial databases (www.trialscentral.org; www.controlled-trials.com, clinicaltrials.gov). Gray literature will be searched in Open Gray and Google Scholar. The reviewers will also screen the reference list of the studies included in the review for additional eligible studies not retrieved by the search.

The search strategy will use a combination of Medical Subject Headings and “entry terms.” The search terms will be divided into four components: population, intervention, outcomes, and study design components. The PubMed search strategy is shown in Table 1.

**Table 1:** Search strategy applied to PubMed.

**Terms**	
1. Population	“postmenopausal” [MeSH Terms] OR “postmenopausal women” [MeSH Terms] OR “menopausal genitourinary syndrome” OR “vaginal atrophy” OR “vulvovaginal atrophy”
2. Intervention	“laser” [MeSH Terms] OR “laser therapy” [MeSH Terms] OR “vaginal laser therapy”
3. Outcomes	“pH” [MeSH Terms] OR “dyspareunia” [MeSH Terms] OR “itching” [MeSH Terms] OR “burning” [MeSH Terms] OR “dysuria” [MeSH Terms] OR “urinary tract infections” [MeSH Terms] OR “urinary frequency” OR “urinary incontinence” [MeSH Terms] OR “vulvovaginal atrophy”
4. Study design	“controlled clinical trials” [MeSH Terms] OR “quasi-randomized studies” **1 AND 2 AND 3 AND 4**

*MeSH, Medical Subject Headings*.

### Study Selection and Data Extraction

The initial selection of titles and abstracts will be performed independently by three authors (LLMNP, APFC, and KSM). Any disagreement will be resolved by consensus. All articles found through the search strategy will be exported to the Rayyan QCRI application for initial selection by considering their titles, abstracts, and keywords.

The full text of the eligible studies will be retrieved for a detailed reading. Two authors (LLMNP and ACAS) will independently examine the full-text articles for compliance with the eligibility criteria. Other studies in the references of the articles included in this review can also be researched. Any disagreement between the reviewers over the eligibility of studies will be resolved through discussion and consensus. When no resolution is reached, a third reviewer (RNC) will be involved in the decision.

**Figure 1:**
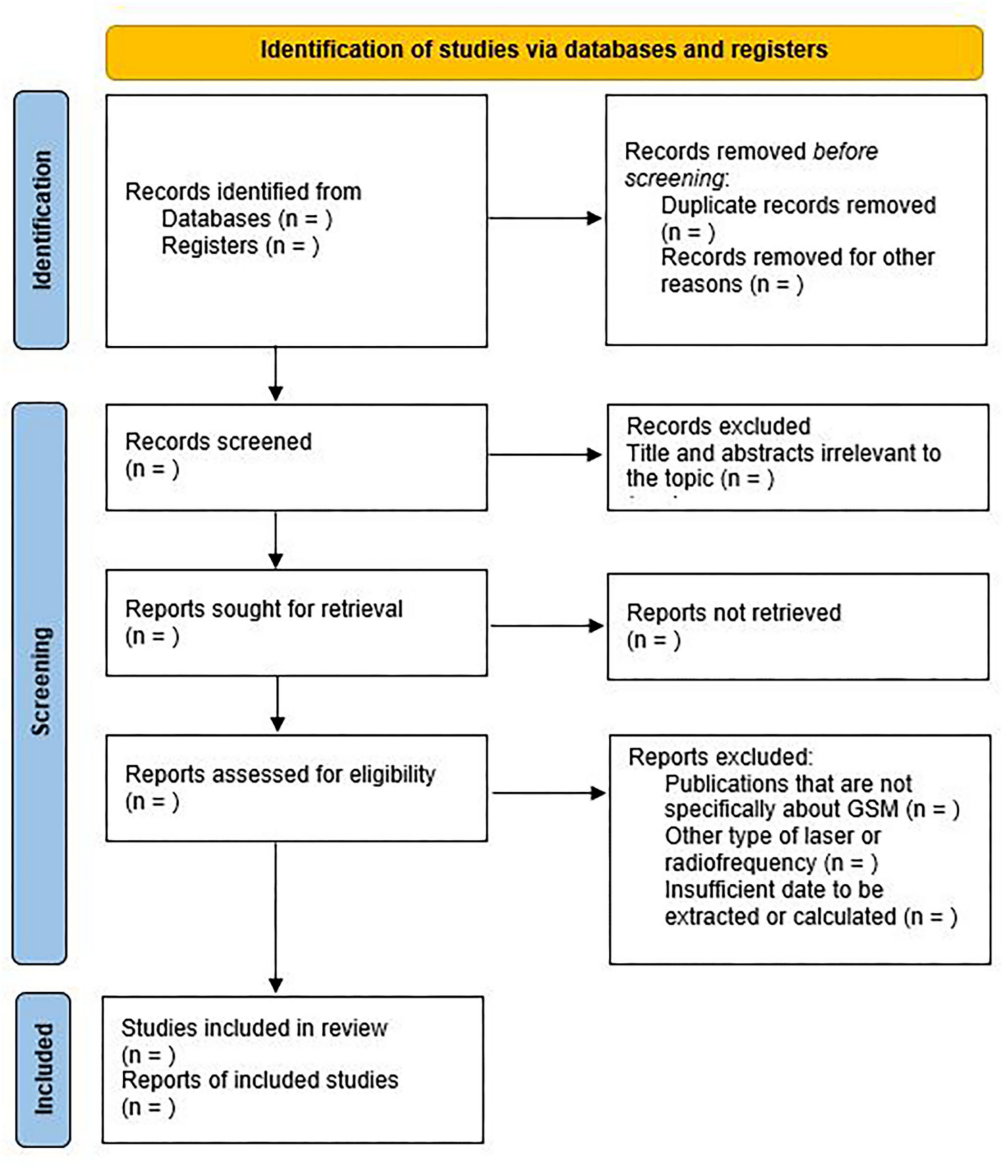
PRISMA flow diagram for systematic review and meta-analysis.

A standardized form, created previously by the reviewers, will be used for data extraction. Missing or additional data for a particular study will be requested from the corresponding author via email by the reviewers (maximum of two attempts). A record of the reasons for excluding studies at all stages of the review will be maintained. The results of the selection or exclusion of studies will be reported using the PRISMA flowchart, as shown in Figure 1.

### Data Extraction and Management

Three review authors (LLMNP, KSM, and ACAS) will independently assess and extract the data of eligible studies, including author, country, type of study, follow-up, mean age, sample size, interventions, and outcomes. Extracted data will be checked by RNC, and any disagreement will be resolved through discussion.

### Addressing Missing Data

If any selected article has insufficient information, the corresponding author will be contacted via email or phone to make a request for the missing data. If retrieval of the missing data is not possible, the data will be deleted and discussed in the section Discussion.

### Risk of Bias Assessment

The risk of bias for each study included will be assessed by two independent review authors (LLMNP and ACAS) using the ROBINS-2 tool (16). This tool allows assessment of allocation (random sequence generation and allocation concealment), blinding of participants and personnel, blinding of outcome assessors, incomplete outcome data, selective reporting, and other biases. Each criterion will be assigned high, low, very low, or unclear risk of bias values. Disagreements will be resolved through a discussion with the contribution of a third author (RNC).

We will construct funnel plots to evaluate publication bias if more than 10 clinical trials are included in the review. Otherwise, Egger's test will be used.

### Assessment of Heterogeneity

The heterogeneity between the studies will be verified using the Cochran's Q test and quantified through *I*^2^ statistics, with values >50% representing high heterogeneity. Meta-analysis will be conducted when at least two studies match the eligibility criteria of the review.

### Strategy for Data Synthesis

The data will be carefully evaluated and extracted from all eligible studies. Data retrieved from the studies will include the first author, year of publication, study design, type of treatment, number of participants, baseline characteristics of post-menopausal women, therapeutic protocol, follow-up period, and measurements of GSM symptoms and outcomes.

For continuous outcome data, a meta-analysis will be performed using the standardized mean difference and 95% confidence interval (CI), calculated by subtracting the mean of the control group from the mean of the treatment group, and dividing the obtained difference by the pooled standard deviation of the two groups. The treatment effect of the binary outcome data will be summarized using risk ratios with a 95% CI. This quantitative synthesis will be performed in the RevMan 5.3.5 software using the inverse variance method with the fixed or random effects model if more than 50% heterogeneity is identified among studies.

In case there are insufficient data to calculate an estimated effect, a narrative synthesis will be presented, describing the direction and size of the effects, along with any reported accuracy measures.

### Subgroup and Sensitivity Analysis

If sufficient data are available, a subgroup analysis will be performed based on the type of laser therapy. If a significant difference between subgroups is identified (test for interaction, *p* < 0.05), we will report the results for individual subgroups separately. We will also perform a formal test for subgroup interactions using the RevMan software.

Sensitivity analysis will be conducted between studies with low and high risk of bias. We will include high risk of bias studies in a secondary analysis to assess the impact on the results. In case of a significant difference between the estimates of the effect of the primary analysis and sensitivity analysis, we will perform an adjusted sensitivity analysis.

### Grading Quality of Evidence

The quality of evidence for all outcomes will be assessed using the Grading of Recommendations Assessment Development and Evaluation (GRADE) approach to evaluate the strength of evidence of the SR results. This approach classifies studies as high, moderate, low, or very low certainty of evidence (17).

## Discussion

Laser therapy has been proposed as a treatment to reduce GSM symptoms, especially in women who refuse hormonal therapy or are at a high risk of complications if they undergo hormonal therapy. However, studies evaluating the effectiveness and safety of laser treatment for GSM have shown controversial results. The authors of two recently published reviews stated that although laser therapy for the treatment of the symptoms of GSM appears promising, there is currently a lack of high-level and long-term evidence (3, 8).

A retrospective, multicenter study involving six hundred forty-five women was conducted after collecting data from a pre-existing database to assess the efficacy and effectiveness of CO_2_ laser therapy in post-menopausal women with clinical signs and symptoms of GSM. The authors concluded that the CO_2_ laser system is effective and tolerable (18). However, Mounir et al. highlighted that the literature regarding vaginal laser therapy in the treatment of GSM is limited to prospective case series with small numbers and short-term follow-up, and high-quality data describing the safety, benefits, and appropriate use of vaginal laser therapy is lacking (19).

On July 30, 2018, the US Food and Drug Administration (FDA) released an FDA Safety Communication stating that “the safety and effectiveness of energy-based devices for treatment of these conditions has not been established” and warned that “the treatment of these symptoms or conditions by applying energy-based therapies to the vagina may lead to serious adverse events, including vaginal burns, scarring, pain during sexual intercourse, and recurring/chronic pain” (20). In the same year, the American College of Obstetricians and Gynecologists in its Position Statement noted that preliminary observational data showed some potential benefits with the use of this technology in treating patients with vulvovaginal atrophy. However, these observational trials did not evaluate the use of concomitant treatments, and they lacked long-term follow-up. Thus, they concluded that additional data are needed to further assess the efficacy and safety of this procedure in treating vulvovaginal atrophy, particularly for long-term benefits (21).

Therefore, a SR with meta-analysis of randomized clinical trials in which laser efficacy and safety are assessed in women with GSM is justified due to the lack of high-level evidence in the scientific literature. With the rigorous methodology that we present in this protocol, we hope that the results of this study will allow healthcare professionals to choose this treatment option with a scientific basis and that they will serve to assist physicians and patients in the informed decision-making process. However, a possible limitation of the proposed study is that clinical trials with a small number of participants, events, or both, leading to wide confidence intervals and high uncertainty of the estimated effects can compromise the level of evidence generated in the meta-analysis.

The SR findings will provide highly relevant evidence through the synthesis of well-designed and robust clinical trials on the effectiveness and safety of laser therapy in GSM.

## Ethics Statement

This study will be a review of published data. Thus, it was not necessary to obtain ethical approval. The findings of this systematic review will be published in a peer-reviewed journal. Patients and/or the public were not involved in the design of the study. Furthermore, they will not be part of the conduct, reporting, or dissemination plans of the study. Therefore, patient consent for publication was not required.

## Author Contributions

LP, KM, AS, and AC contributed to the design of this review. LP, KM, and AS drafted the protocol manuscript, and RC and AG revised it. LP, KM, and RC developed the search strategies, and LP, KM, and AS will implement them. LP, KM, AS, and AC will track potential studies, extract data, and assess quality. In cases of disagreement between the data extractors, RC will advise on the methodology and work as a referee. All authors approved the final version for publication.

## Funding

This study was financed in part by the Coordenação de Aperfeiçoamento de Pessoal de Nível Superior - Brasil (CAPES) - Finance Code 001.

## Conflict of Interest

The authors declare that the research was conducted in the absence of any commercial or financial relationships that could be construed as a potential conflict of interest.

## Publisher's Note

All claims expressed in this article are solely those of the authors and do not necessarily represent those of their affiliated organizations, or those of the publisher, the editors and the reviewers. Any product that may be evaluated in this article, or claim that may be made by its manufacturer, is not guaranteed or endorsed by the publisher.
